# Case Report: Introducing @tension—a system for improving adherence to exposure therapy in chronic pain patients

**DOI:** 10.3389/fnhum.2026.1708612

**Published:** 2026-02-11

**Authors:** Shifra Berkowitz, Keren Sivan Speier, Gadi Bartur, Hadassah Fortinsky, Bracha Auerbach Lawee, Goded Shahaf

**Affiliations:** 1 Reuth Medical Center, Tel Aviv-Yafo, Israel; 2 Rambam Health Care Campus, Haifa, Israel

**Keywords:** adherence, case reports, chronic pain, exposure therapy, monitoring

## Abstract

Exposure therapy is an effective treatment approach for chronic pain that depends on patient adherence. However, it may evoke fear of pain and even flare-ups, which may lead to non-adherence to the therapy. More specifically, the @tension (pronounced At tension) system aims to provide “protected” graded exposure to assist patients who might otherwise not adhere to exposure therapy. @tension includes real-time monitoring of the patient’s discomfort and attention during graded exposure therapy, and provides online feedback indicating potential increases in discomfort due to pain or stress, which may indicate a good time to pause the exposure for a while, and then resume practice once the patient is ready again. After initial advancement, patients are encouraged to continue the exposure without pauses and to employ techniques to calm the evolving pain. The purpose of this article is to present the @tension system and to demonstrate its potential for assisting patients with chronic pain using representative case reports of patients who encountered difficulties adhering to exposure therapy before using the system. We believe the @tension system may be of potential interest to clinicians and researchers in the field and will be happy to provide the software and needed support.

## Introduction

Exposure therapy is a prevalent treatment approach for chronic pain of various types and appears effective when patients adhere to the therapy (CRPS – den Hollander et al., 2016; Fibromyalgia – Hedman-Lagerlöf et al., 2018; Low back pain – Glombiewski et al., 2018), especially when combined with other interventions, such as pain education (Marris et al., 2021). The exposure is often performed in a graded manner to improve patients’ adherence (Woods and Asmundson, 2008). Less gradual exposure has been advocated to enhance treatment efficacy (den Hollander et al., 2022). However, in many cases, it can actually increase fear of pain and even evoke flare-ups (Kroll, 2015), which may lead to non-adherence to the therapy (Turk and Wilson, 2010).

We have been developing an easy-to-use computer system called @tension (pronounced At tension) for enabling “protected” graded exposure to assist patients who might otherwise not adhere to exposure therapy due to fear of pain. The @tension system includes real-time monitoring of the patient’s levels of discomfort and attention during graded exposure therapy. The system provides online feedback to the patients regarding potential increases in discomfort due to pain or stress, which may indicate a good time to pause the exposure for a while, and then resume practice once they are ready again. As patients progress, they are encouraged to continue the exposure while employing interventions to calm the evolving pain during the task. Ultimately, patients may learn to internalize the avoidance of over-exposure and the calming of evolving pain even without the system. The system supports self-practice by the patients at their convenience in their preferred environments. After the session, therapists can review the data and provide tele-recommendations toward the next sessions.

The system is based on two electrophysiological markers, measured from a single forehead EEG channel. Over the last decade, we have been developing real-time markers for attention-related processes. The efficacy of our markers has been demonstrated by us, as well as by multiple other independent research groups, using thousands of samples from multiple clinical populations (Shahaf et al., 2018b; Keren et al., 2024; Isserles et al., 2018; Shahaf et al., 2018a; Gvion et al., 2021; Vasquez et al., 2023; Yogev-Seligmann et al., 2022; Bart and Liberman, 2020; Baron Shahaf and Shahaf, 2024; Baron Shahaf et al., 2022). Furthermore, our markers have been implemented in multiple rehabilitation protocols (Bartur et al., 2020; Karpin et al., 2022; Gvion and Shahaf, 2023). The first marker in the @tension system is the TensI (tension index) marker for alertness, which is used for monitoring discomfort (Baron Shahaf and Shahaf, 2024). The second marker is the cognitive effort index (CEI), which is used for monitoring sustained attention (Baron Shahaf and Shahaf, 2024; Baron Shahaf et al., 2022; Gvion and Shahaf, 2023; Keren et al., 2024).

The purpose of this article is to present the @tension system and to demonstrate its potential for assisting patients with chronic pain through representative case reports of patients who encountered difficulties adhering to exposure therapy prior to using the system.

## Methods—the constituents of the @tension system

The underlying TensI and CEI markers (for further details see Baron Shahaf and Shahaf, 2024) are extractable from every single EEG channel with minimal adaptations of the interface (Baron Shahaf and Shahaf, 2024; Baron Shahaf et al., 2022; Gvion and Shahaf, 2023; Keren et al., 2024). In this report, the BrainLink mobile headset^1^, which is one of multiple headset types that embed the core ThinkGear integrated circuit^2^, is used.

### Computation of the CEI (cognitive effort index)

The EEG sample is divided into 10-s segments. Each 10-s segment is filtered to the delta band (1–4 Hz), and then the filtered segment is divided into 20 epochs of 500 milliseconds each. For each epoch, the power of the delta activity is computed, and then the mean and standard deviation of all epochs within a segment are calculated. Then the CEI is derived from the standard deviation to mean ratio and is in the [0,1] range. We found that if the ratio is >1, it is likely due to a noisy sample, in which case no value is returned for that 10-s segment. Our experience with the CEI indicates that values in the middle third range (between 1/3 and 2/3) represent an effective attentional effort, while values below 1/3 may represent too low effort, and values above 2/3 may represent attention to stressing stimuli (see more details in Gvion and Shahaf, 2023).

### Computation of the TensI (tension index)

For each 10-s segment, we compute the power of high beta activity (23–30 Hz). Segments in which the CEI value is in the middle third are considered baseline segments, and the mean baseline beta power of these segments is updated with every additional baseline segment during the sample. The TensI value for a given segment is derived from the ratio between the power of beta activity in the given segment and the mean beta power of the baseline segments preceding it. The TensI value is this ratio divided by two, so that when the current segment beta power is equal to the mean baseline beta power, is 0.5. If the TensI is greater than 1, it is set to 1. Thus, the TensI is also in the [0,1] range. The specification of baseline activity, according to segments with the CEI in the middle (effective attentional effort range), accords with other researches (Palacios-García et al., 2021).

The approach of excluding segments in which the standard deviation is greater than the mean reduces overt noise due to intense muscle activity (Baron Shahaf and Shahaf, 2024). However, especially when prefrontal recording is considered, there is always concern regarding the impact of “milder” EMG noise sources. There seems to be a range of overlap in which it is uncertain whether activity originates from the brain or from muscle activity. Specifically, for the CEI and the delta activity in this region, there is major concern regarding the effect of blinking (Delorme et al., 2007). However, interestingly, blinking is well-related to attention (Maffei and Angrilli, 2018). Furthermore, the pattern of “attentive” well-deferred blinking in the delta bandpass may lead to greater variability of the signal, which is captured by the CEI marker (Baron Shahaf and Shahaf, 2024). Therefore, we did not see a practical need to differentiate between EEG activity and blinking. For the TensI and high beta activity in this region, there is major concern regarding the effect of the frontalis muscle contraction (Goncharova et al., 2003). However, interestingly, increased frontalis contraction is related to stress (Orr et al., 1993), which is captured by the TensI marker. Therefore, there is no practical need to differentiate between EEG activity and frontalis contraction.

#### Identifying deviations in alertness (TensI) or in sustained attention (CEI)

**Figure 1:**
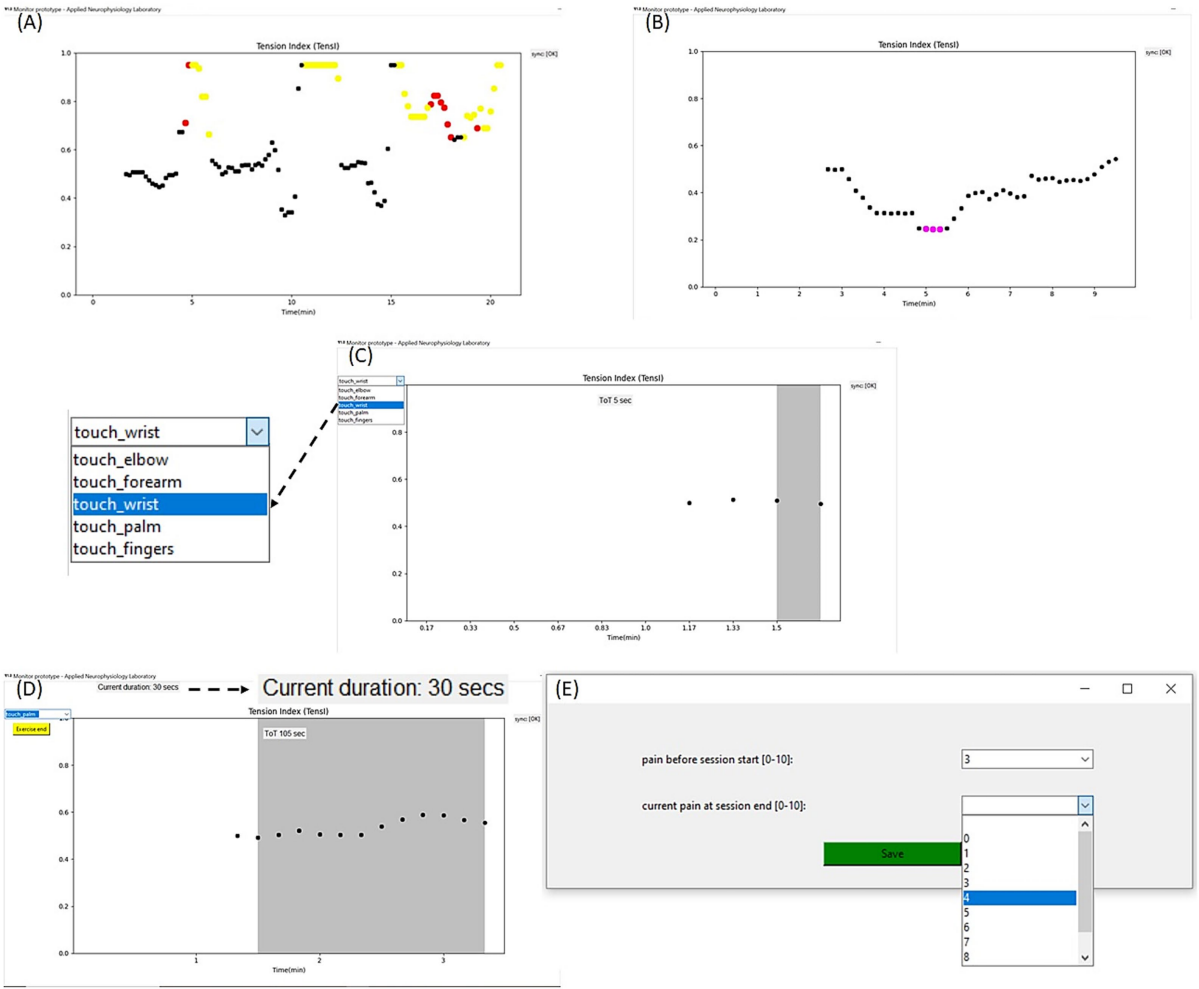
Visualization of the @tension system. **(A)** Increase in TensI above an operator-defined high threshold (not shown) may indicate an increase in pain or stress. The values are then marked in either red, if the simultaneous CEI value is outside the effective middle range, which may indicate also impaired sustained attention, or in yellow, if the CEI value is within the middle range, which may indicate effective sustained attention, despite the pain or stress. The CEI values are not shown by the system. **(B)** Decrease in TensI to below an operator-defined low threshold (not shown), with a simultaneous CEI out of the middle range, may indicate avoidance from attending to the exposure task, as may occur in a dissociative response. These TensI values are colored in pink. The CEI values are not shown by the system. **(C)** Levels of exposure are predefined by the patient and the therapist. Each task in the session starts by pressing a green button (not shown), and then the list of levels of exposure opens and the recommended level of the task is marked, but could be changed. The gray background marks the task on the graph. **(D)** TensI values are presented every 10 s. For each task duration, which is marked by the gray background. The tens of seconds in which there is no deviation of the TensI value, above the high threshold or below the low threshold, and also the CEI value (not shown) is in the middle effective range, are summed and presented as “current duration” near the top left corner. **(E)** End of session collection of data regarding the pain at the beginning of the session and at the end of the session reported by VAS, as one of the factors, which are considered by the guiding therapist, when deriving recommendations for the next sessions.

If the TensI marker increases above a high (operator definable) threshold, the values are colored to indicate a suspected increase in pain or stress. A TensI value is colored in red if the simultaneous CEI value is outside the middle range, which may also indicate ineffective sustained attention, or yellow if the CEI value is within the middle range, which may indicate maintenance of effective sustained attention despite the possible discomfort (Figure 1A). If the TensI marker decreases below a low (operator definable) threshold, and the CEI is outside the middle range, indicating a simultaneous ineffective sustained attention, the TensI value is colored in pink (Figure 1B). This may indicate avoidance of attending to the exposure task, for instance, in a dissociative response. Note that only the TensI values are presented by the system, while the CEI values affect the coloring as specified but are not presented, as we found it is advisable to avoid information overload. The @tension system may produce recorded audio verbal alerts during the session, when the various thresholds are crossed and the TensI values are colored, according to specific operator preferences, so the patient does not need to attend to the screen.

#### The specification of levels of exposure

As seen in Figure 1C, the therapist, together with the patient, defines a desired functional goal as the long-term target level (example from the figure: “touching the fingers”). For each functional goal, an initial level is defined, which is currently feasible with some effort (example from the figure: “touching the elbow”), along with intermediate exposure levels that together create the span of an exposure protocol. Each patient can set several desired functional goals and their exposure protocols (e.g., a sensory goal and a motor goal), as long as the patient allocates enough time to practice these multiple exposure protocols. Once a long-term goal is obtained, it is possible to set a new, more demanding long-term goal, and if relevant, to start the new protocol with this recently obtained level as the new initial level.

#### Recommendation to advance through levels of exposure

During the session, the patient presses a button to start each task, which is then marked with a grey background on the TensI graph. During the task, every 10 s in which the TensI marker does not deviate, and the CEI marker (not shown on the graph) remains in the middle and effective range is counted and presented at the top left of the computer screen as the effective duration in the current task (Figure 1D). If this score crosses an operator-definable threshold by the end of the task, the system advises the patient to advance to the next level of exposure according to the pre-specified levels. This is merely a recommendation, and the patient can choose to select the level of exposure manually, as noted in Figure 1C.

#### Measuring the impact of the session on the level of pain

The goal of the @tension system is to support protected exposure, and for this purpose, it is important to try avoiding exposure-induced flair-ups. Therefore, as part of the data considered by the guiding therapist, the patient is asked to report the VAS pain score both at the start and end of the session (Figure 1E).

#### Concise guidance can be provided as a post-session tele-feedback

After the session, the therapist receives the session data file from the patient and reviews it to provide guidance for the next session. The therapist may recommend adjustments to the exposure, tune the thresholds by which the system alerts regarding deviations in the TensI and CEI markers, change the required duration of effective exposure before a recommendation to advance is made by the system, and recommend changing the exposure levels or adding new functional goals for exposure. The therapist usually takes only a couple of minutes to evaluate the session data and send feedback. Thus, for each therapist’s treatment hour, patients can receive feedback for multiple sessions performed in a convenient setting.

## Results–case presentations

### Two types of patients who may need assistance: the over-exerting and the under-exerting

Studying the potential usefulness of the @tension system with several dozen patients, our interim conclusion is that it may be beneficial for patients who have major difficulties with adherence to a systematic exposure therapy. Thus far, there appears to be two types of patients who may be able to adhere to the therapy better with the system than without it. The first type are patients who over-exert when practicing exposure therapy, to the degree that the post session pain or stress becomes intense and may even lead to flare-ups lasting for hours or even days. Consequently, these patients often fail to adhere consistently to the therapy. On the other hand, the second type are patients who experience significant fear of pain during exposure therapy, leading to avoidance of overall exposure. In this initial presentation of the @tension system, representative patients from each of these two groups are described to demonstrate the potential usefulness of the @tension system for both the over-exerting patients and those who under-exert due to fear of pain. While patients who may under-exert due to fear of pain often require long interventions with the system, our experience indicates that some over-exerting patients can learn more quickly, using the system for only a few sessions, not to over-exert, and can continue implementing this even without the system. Patients who under-exert due to fear of pain may eventually learn to continue the exposure without the system, but this process is often significantly quicker with over-exerting patients. Therefore, two over-exerting patients are presented: one who used the system for multiple sessions at home with tele-guidance, and another who required only a few sessions at the clinic. Thereafter, a patient who under-exerted due to fear of pain and used the system for multiple sessions at home with tele-guidance is described.

The diagnosis of each patient’s clinical condition was made by a multidisciplinary team of experts, led by a pain medicine doctor and a psychiatrist at Reuth rehabilitation center that functions as a national diagnostic and treatment hub for complex regional pain syndrome (CRPS). CRPS was diagnosed in accordance with the Budapest criteria, and psychiatric conditions (PTSD, depression and somatization) were diagnosed in accordance with DSM-5 criteria. The patients continued their other treatments without change throughout the period of @tension treatment, except for the change induced in the exposure therapy by use of the system. Continued treatment included physical therapy, which focused on transfers and mobility with aids such as crutches and wheelchair, as well as biofeedback and psychotherapy, each of the treatments were initiated at least 4 weeks prior to the start of the @tension treatment.

### The first patient held back by over-exertion who has been using the system for multiple sessions

#### Clinical presentation

Patient N is a 24-years-old female with an unremarkable medical history. She was referred to rehabilitation as an outpatient in our institute 6 months after she was diagnosed with CRPS type 1, following a limited tear of the right anterior talofibular ligament during a misstep on a field trip, and was treated conservatively. Patient N abstained from using chronic pharmacotherapy. Prior to her referral to working with the @tension system, she underwent 3 months of standard rehabilitation with little progress. When she started working with the @tension system, she had very limited weight-bearing ability on the injured leg, and was unable to stand or walk for more than a few seconds without the use of crutches. The ankle and the foot were edematous, purple, and very sensitive to touch according to anamnesis and medical evaluations. She suffered severe bouts of pain (reaching a VAS 10 from a baseline of VAS 6) within seconds of wearing a shoe or sock, sleeping with a blanket on her foot, washing her foot with a direct water flow, or cutting her fingernails. She appeared motivated to improve, possibly even over-motivated, because prior to using the @tension system, she ended multiple exposure sessions with her occupational therapist and physiotherapist with exacerbation of the pain that often lasted for hours. This led to very limited self-practice.

#### Monitoring to assist in pausing exposure before an intense increase in pain

**Figure 2:**
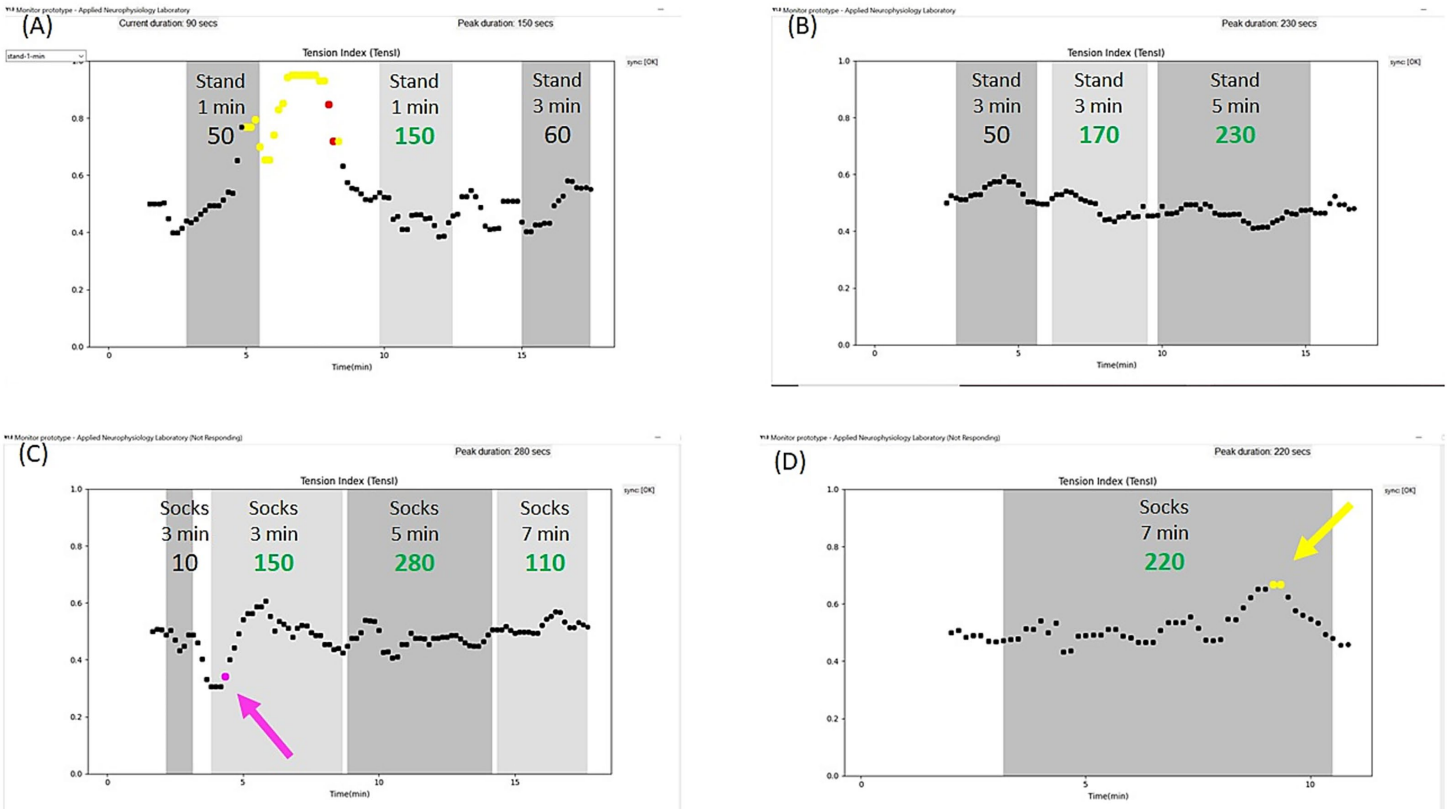
Sessions of Patient N. **(A)** Patient N practiced standing without crutches. The first level of exposure was to stand for one minute, rest for one minute, stand again for one minute, etc. She was encouraged to pause and take a recess if she felt an evolving pain, to avoid a lasting pain. In the first task, marked with dark grey background on the left, she did not stop on time, as could be seen by the increase in the TensI marker and the change to yellow before the end of the grey marking of the task. At this stage, the system sounded an audio verbal alert suggesting to pause the current task (stopping the task is marked by pressing a button – not shown). The patient was then asked not to start a new task immediately, but to relax and wait for the TensI values to decrease to below the high threshold, turning black again. Thereafter, she started the task again, as is marked by the light grey background (dark and light grey backgrounds are used interchangeably). This time she managed to stay in the effective range of TensI and CEI (not shown) for 130 s (the “effective duration” of each task is presented below the task name), which was above the predefined threshold (120 s) and it was recommended by the @tension system audio verbal feedback to advance to the next level of exposure (effective durations above threshold are typed in green). The patient accepted the recommendation and therefore the task, marked with the right dark grey background, was already of the second level of exposure – standing for 3 min. **(B)** Patient N continued to advance among the predefined levels of exposure. Here we can see that she stopped on time, prior to the exacerbation of pain, in the first (left) task of 3 min standing, then exceeded the predefined threshold of 120 in the second (middle) task. Then, after advancing to the next level of exposure of 5 min standing, she also exceeded the predefined threshold, in the third (right) task and the next session (not shown) started at the next level of exposure – 7 min standing. **(C)** Patient N started soon also exposure for additional functional goals, and in this case, wearing socks. She also started to practice soothing an evolving pain during the task without stopping the exposure. Following guidance from her occupational therapist, she focused on her breathing and on the task itself rather than on the evolving pain. We often start with continuing the task despite a decrease in the TensI to below the low threshold, combined with a deviation of the CEI (not shown) from the effective range, which is marked in pink, plausibly denoting avoidance and a possible dissociation. As can be seen in the figure, she managed to continue, with resumption of effective markers (only TensI is shown), without pausing the task. **(D)** Patient N also succeeded in continuing the task and resuming effective markers when the evolving pain involved an increase in the TensI marker to above the high threshold (marked in yellow), despite the increase in pain.

When Patient N started to work with the @tension system, the first goal was learning to pause the tasks during the session before an exacerbation of the pain, to prevent a lasting post-exposure pain episode. Our experience with patients who tend to over-exert is that the @tension system helps them to rapidly learn to respond to early signs of pending pain or stress exacerbation during exposure, and to pause the tasks on time to avoid a lasting exacerbation. As demonstrated in Figure 2A, this goal may be accomplished within a single session. For patient N, pausing the exposure before an intense increase in pain prevented post-exposure exacerbations. This rapid learning often leads to compliance with the exposure therapy, under the guidance of the @tension system, as patients feel more protected. Thereafter, compliance often extends to moderating exposure even without the system, as the patient learns to rely on self-monitoring for stopping before exacerbation. Figure 2A also shows, how after taking time to relax the pain, Patient N advanced from one minute standing to 3 minutes (right grey region). The alleged “slow down” due to pausing exposure while experiencing increased pain or stress does not prevent progress. Resuming the exposure a few minutes later, when the patient is more relaxed, may enable more effective exposure. Thereafter, as shown in Figure 2B, Patient N continued to progress swiftly.

#### Monitoring for assistance with continuing exposure tasks despite some pain

Patient N progressed almost daily with multiple functional goals. She reported a feeling of greater control and significantly less fear of pain, but still preferred the monitoring provided by the @tension system. She also started to work on attempts to calm an evolving pain during tasks without stopping the exposure. She practiced focusing on breathing and on the task at hand rather than on the pain. Thresholds of the @tension system can be reset to provide the audio-verbal alerts if the deviation of the TensI marker exceeds a definable time duration. We often start the next phase of continuing the task, despite some limited pain, by first trying to continue the exposure despite an avoidance or a dissociative response, depicted by a TensI decrease and ineffective CEI (not shown), marked in pink (Figure 2C). Then, when the patient feels ready, we advance to practicing continued exposure despite TensI increases, which indicate increased pain or stress. These are marked in yellow when CEI is in the effective range or in red when CEI is outside the effective range (Figure 2D). Patient N gradually learned to master the ability to continue the exposure despite pain onset over weeks of exposure practice.

#### Expanding to ADL without the system

Baseline pain (VAS 6) did not change. However, she regained most of the daily functions that she had difficulties with for months, including standing and walking without crutches, wearing a sock and shoe, sleeping with a blanket, washing her foot, and cutting her fingernails, with about 90% reduction in the number and intensity of bouts of increased pain. After several weeks, she felt confident to monitor both her exposure sessions and her daily activities without the @tension system. Altogether, Patient N used the @tension system at home nearly daily and sent computerized session reports to her therapist after each session for 10 weeks.

#### Major formal start vs. end assessments (physical disability)

10-meter walk: start – 12 s with crutches, end – 9 s without crutches; 6-min walk: start – 400 meters with crutches, end – about same distance without crutches; stair climb test: start – 21 s with crutches, end – 15 s without crutches; allodynia: start – at the ankle region, end – normal sensation.

### The second patient also held back by over-exertion, but only needed the system for a few sessions

#### Clinical presentation

Patient L is a 35-year-old female with an unremarkable medical history. A year prior to her admission for rehabilitation as an outpatient in our institute, she sprained her left ankle during a leisure sport activity. Initial suspicion of a navicular bone fracture was refuted by an MRI, which did not demonstrate any major traumatic findings. However, she has been suffering from intense pain and tended to abstain from standing without crutches. She could also not tolerate even a light touch to her foot or ankle region, which were edematous and purple-blue in color according to anamnesis as well as in medical evaluations. She refrained from applying a direct water current to her foot, and could only cut her fingernails after taking Oxycodone. Following the orthopedic and pain workup, her working diagnosis has been CRPS type 1. Over the last couple of months, the pain and sensitivity spread gradually toward the knee. Her baseline VAS was 6–7, with severe bouts of increase to 9 and 10. For the last couple of months, she has been taking Oxycodone as an SOS upon such pain exacerbations, nearly daily, but did not want to use other chronic medications. Prior to working with the @tension system, she had undergone near-daily physical therapy and occupational therapy exposure sessions for 6 weeks without significant progress. During this period, she frequently reported exacerbation of the pain following the sessions, often for hours on end. This led to very limited self-practice.

#### Swift learning to pause exposure before an intense increase in pain

**Figure 3:**
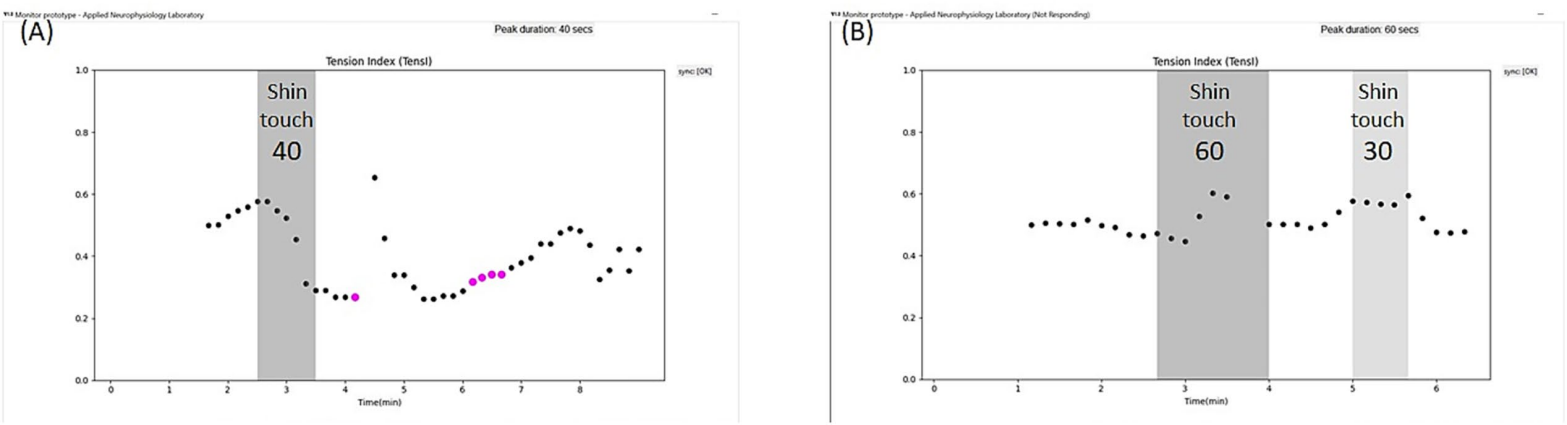
Sessions of patient L. **(A)** Patient L practiced touching her left leg, starting at the feasible shin level. The goal was to learn to stop on time, when discomfort and pain only start to increase, in order to avoid lasting pain. In the first session, after less than a minute into the task, TensI dropped, possibly indicating avoidance and dissociation, and the patient was encouraged by her occupational therapist to consider taking a break. She stopped and shared that she felt the increase in pain, but planned to continue. Her occupational therapist pointed out that this approach led to a lasting increase in pain and did not result in progress. During this conversation, the TensI was still low, but with only a few pink points, which means her CEI (not shown) and possibly her sustained attention were mostly effective. She was also encouraged to finish the session whenever she felt the pain increase and to resume it after relaxation, and so she started with sessions of only a few minutes. **(B)** In the following session, she managed to stop the tasks on time without deviation of the TensI marker, and she also reported that she started practicing at home repetitively while stopping on time and avoiding significant increases in pain. Then, in the third session (not shown) she managed to touch not only her shin, but her foot already without using the system, after practicing previously at home with stopping on time. The use of the system was discontinued, and she continues to improve with different tasks.

When Patient L started working with the @tension system, the first goal was also learning to pause the tasks during the session before an exacerbation in pain, to prevent a lasting post-exposure pain episode. In accordance with our experience with multiple patients as described above, patient L learned swiftly, within a couple of sessions, to pause the task and avoid exacerbation in pain (see Figure 3). In between these two sessions, she practiced repetitively without the system at home to pause the tasks prior to pain exacerbation, and managed to master the process. She continued with repetitive self-practicing and pausing prior to pain exacerbation following the second session, and started to advance among exposure levels. When she demonstrated how she advanced to touching her foot by the third session, her use of the @tension system was discontinued, as she felt confident she could progress effectively also without it.

#### Advancing constantly following the short use of the system

We are now a few weeks after the three sessions in which she used of the @tension system. Patient L started to stand and walk without crutches for a few minutes at a time. She manages to wear a shoe on her left foot for a few minutes at a time and is consistently undertaking more household chores and activities with her children, while gradually resuming social interactions. She reports that in the vast majority of these tasks and activities, she feels in control of the situation and takes pauses when she feels pain may increase beyond a manageable level. She expresses optimism regarding her ability to continue progressing.

#### Major formal start vs. end assessments (physical disability)

10-meter walk: Start – 1 min with crutches, end – about the same time without crutches; 6-min walk: start – 50 meters with crutches, end – 70 meters without crutches; allodynia: start – from the middle shin and below, end – only at the foot region.

### A patient held back by extreme fear of pain

#### Clinical presentation

Patient Z is a 30-year-old male with an unremarkable medical history. About a year prior to his admission to rehabilitation as an outpatient in our institution, he was involved in a car accident. His check-up in the emergency department revealed no physical findings, and he was released home. Shortly after that he experienced severe pain in his right leg. The pain increased over the last year and became debilitating, accompanied by intrusive flashbacks from the accident and a depressive mood. Medical evaluations did not disclose significant edema, color change, or temperature abnormalities. He was diagnosed as suffering from PTSD, a severe depressive episode, and somatization expressed through pain. His pharmaceutical treatment included quetiapine, duloxetine, cannabis oil, and pregabalin. He refrained from ADL activities and used a wheelchair for mobility. Due to his condition, Patient Z refrained from social contacts and activities that interested him in the past. In his 4 weeks of sessions with his occupational therapist and physiotherapist, prior to using the @tension system, he was avoidant and did not succeed in practicing exposure due to fear of pain. After these ineffective attempts to engage him in these sessions, he was referred to us. Use of the @tension system in Patient Z was focused on motor and somatosensory graded exposures, as a part of a multidisciplinary team effort addressing his complex emotional status.

#### Monitoring for assisting in very gradual exposure with encouragement to stop when in fear/pain

**Figure 4:**
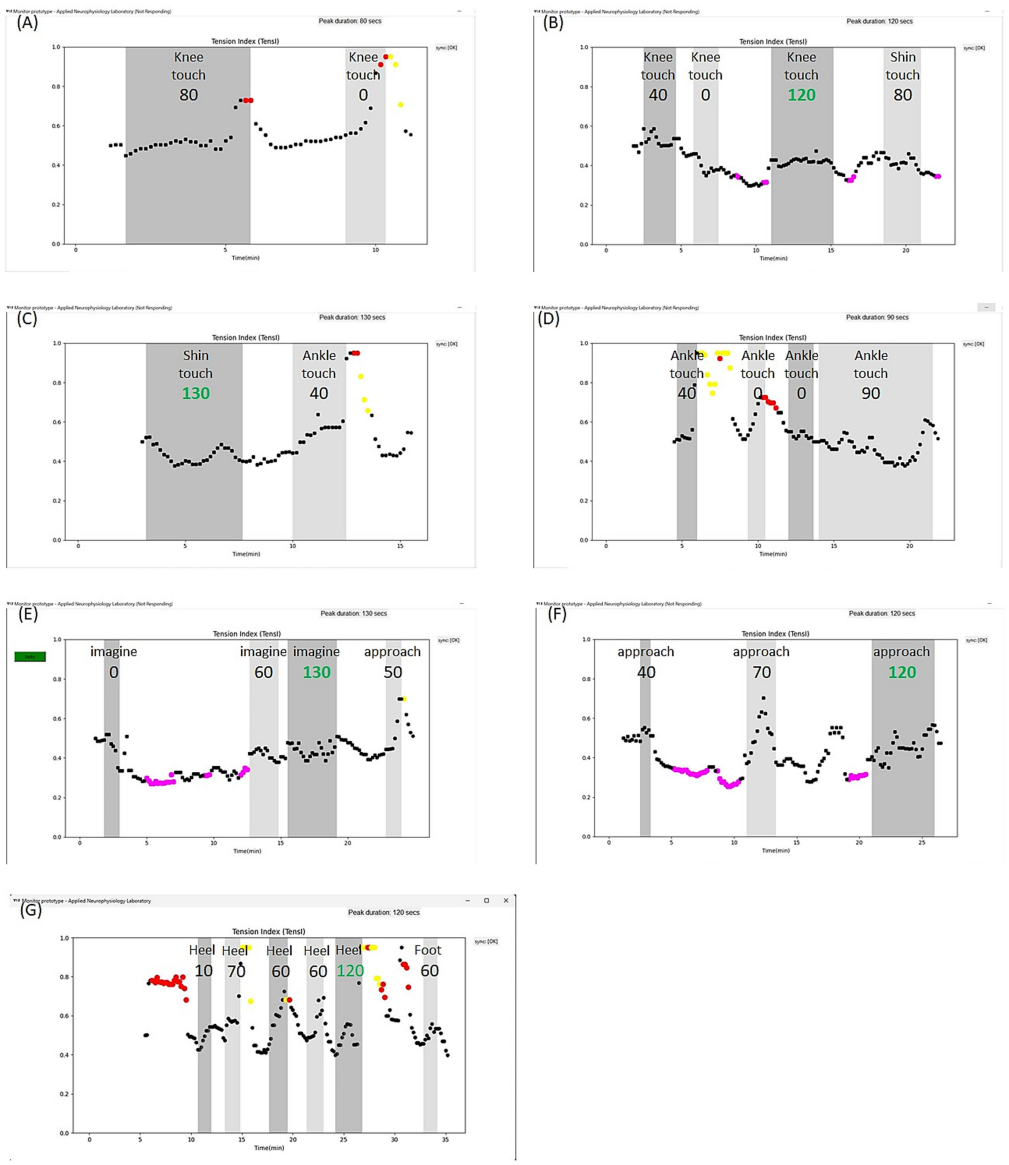
Sessions of Patient Z. **(A)** Patient Z practiced touching his right leg, starting at the feasible knee level. In the first sessions, the goal was to learn that the @tension system offers him protection by pausing before pain or stress exacerbation. The system suggested pausing each task when deviations of the TensI marker were detected, which were mostly increases above the high threshold. **(B–D)** Then, very gradually, Patient Z managed to pass the predefined threshold (≥120 s), in the effective range of TensI and CEI (not shown), for recommendation to advance to the next level of exposure, with touching the knee **(B)** and then with touching the shin **(C)**. The patient has now reached touching the ankle **(D)** and can gradually extend the time in the effective range of TensI and CEI. **(E–G)** Patient Z started working in parallel, and again very gradually, on a motor task of placing his foot on the floor. At first **(E)**, he only practiced imagining the foot on the floor, and after a couple of weeks, managed to pass the predefined threshold (≥120 s), in the effective range of TensI and CEI (not shown), for recommendation to advance to the next level of exposure. Then **(F)** he managed to pass the predefined threshold while lowering his leg toward the floor, without touching it, and he then practiced putting his heel on the floor. After a few weeks **(G)**, he managed to place his heel on the floor and started placing the whole foot on the floor.

During the first sessions with the @tension system, the goal for Patient Z was to feel secure with the system and comply with practice. A low initial level of exposure was selected, and he was encouraged to stop the task as soon as he felt an increase in pain or fear from pain, or when the system alerted him to pause using audio-verbal alerts following the detection of deviation in the TensI and CEI markers. He was pleased to see an objective marker that captured the increases in his pain (Figure 4A). Similarly to other patients, Patient Z encountered responses of doubt from his surroundings, and the objective feedback provided external reassurance. Nevertheless, he was informed that the @tension system is not a polygraph and even if it does not yet capture pain or stress he already feels, he should stop the task to calm before resuming later. He learned that the system provides extra-protection during exposure, and within one or two sessions he was recruited to work with it once or twice daily at home. Thus far, he has used the @tension system for about 3 months, sending computerized session reports to his therapist after each session.

Patient Z progressed slowly through the levels of exposure, which were defined and entered into the system together with his occupational therapist. On average, he progressed one level every 2–3 weeks of practice. This pace may be slow, but it is represents consistent progress and enabled him to engage in the activity (see Figures 4B–D for one functional goal, and Figures 4E–G for another functional goal). Patient Z is still undergoing his long exposure journey with the @tension system and continues to progress toward functional goals. He already tries to relax an evolving discomfort during a task, without stopping, by focusing on his breathing and using other techniques taught to him by his occupational therapist. This is done very gradually, and he still appears to require the protection of the @tension system.

#### Improving general wellness

Following routine use of the @tension system, Patient Z reported a major change in his affect and ADL outside the @tension system sessions. He participates in activities and pauses whenever he feels discomfort. He stated that he feels more in control, whereas previously he felt that the pain was in control. While the change in his objective performance may still be minor, the change in his affect and general attitude seems major. The change was evident in his leisure activity, which changed from staying in bed most of the day, and lack of motivation to undertake any type of activity, just prior to using @tension, to the present state in which he enjoys visiting friends, painting, listening to music, and watching TV.

#### Major formal start vs. end assessments (psychiatric)

Beck depression inventory score (BDI): Start – 42, end – 25; posttraumatic diagnostic scale (PDS): start – 46, end – 32.

## Summary

In this article, we present for the first time the feasibility of the @tension system and its current protocol for chronic pain. The system was developed to provide an extra layer of protection to patients who may not adhere to in-vivo exposure therapy without it. The system operates based on the feedback regarding deviations in indices that monitor effective levels of alertness and sustained attention (Baron Shahaf and Shahaf, 2024; Baron Shahaf et al., 2022; Gvion and Shahaf, 2023; Keren et al., 2024). Our clinical experience with the system, potentially biased but still potentially worthy of sharing, suggests that it may be useful for patients who fail to adhere to exposure therapy, either because they over-exert or because they under-exert due to fear of pain.

The first two cases presented the feasibility of using the @tension system for supporting exposure in patients, who, prior to the system’s use, tended toward over-exposure, which led repetitively to exacerbation of pain to the level of flare-ups of severe pain for hours on end, and to lack of long-term adherence to exposure. Using the system enabled these patients learning rather quickly to identify on their own early signs of pain or stress exacerbation and to pause the task on time in order to avoid lasting exacerbations or flair-ups. In the first case, the patient preferred to continue using the @tension system at home for several weeks, with offline feedback from her occupational therapist, even after learning to identify early exacerbations and to avoid over-exposure. This patient later also used the @tension system to support her ability to calm evolving pain during exposure, potentially by providing external and objective reassurance. For the second patient, a couple of sessions at the clinic with the @tension system sufficed, and she internalized the ability to identify early signs of exacerbation, to pause exposure temporarily, and resume it as soon as she felt ready again. Thereafter, she implemented these abilities in continued exposure sessions.

The third case demonstrated the feasibility of using the @tension system to support exposure of a patient, who, prior to the system’s use, avoided exposure due to a debilitating fear of pain. The patient was reassured to find an objective marker that externalized his major discomfort during exposure. Patients are informed that the @tension system is not a polygraph; it only serves as another layer of protection, and exposure should be paused primarily according to their own identification of exacerbation until they are ready to resume. Nevertheless, the objective marker may have value, considering responses of doubt the patient encountered from his environment. As the patient understood that the system offered another level of protection during exposure, he readily practiced exposure with the system (even more than once daily), which represented a major change. Although the change in his physical disability is still limited, his affect appeared to improve significantly. Experience indicates that working with the @tension system should be assessed and studied only as a “second line” intervention. When patients manage to adhere to exposure therapy without it, they may progress more swiftly (den Hollander et al., 2022). However, many patients may suffer from persistent intense fear of pain or recurrent pain flare-ups following exposure (Kroll, 2015), which may lead to lack of treatment adherence (Turk and Wilson, 2010). In which case the slower and potentially more comfortable mitigation of exposure with the use of the @tension system, at-least for a limited period, may permit slow progress, which would be preferable to no progress at all.

This study is an initial presentation, and even prior to formal studies, there is a need to systematically collect data on adherence to exposure therapy with the @tension system and its ADL and clinical outcomes. However, the system might be of potential interest to other clinicians and researchers in the field, and we will be happy to provide the software and needed support (free of charge for researchers).

## Data Availability

The datasets presented in this article are not readily available because of ethical and privacy restrictions. Requests to access the datasets should be directed to the corresponding author.

## Footnotes

1
https://o.macrotellect.com/2020/BrainLink_Lite.html

2
https://www.seeedstudio.com/ThinkGear-AM-Brainwave-Sensor-p-1441.html?srsltid=AfmBOor8PlvT1qjlEOVnJJ4H8D_W7G4d-6lWvnJkQgGs0zp6kJvq1Qhq

## References

[ref1] Baron Shahaf D. Shahaf G. (2024). Markers of too little effort or too much alertness during neuropsychological assessment: demonstration with perioperative changes. Brain Behav. 14:e3649. doi: 10.1002/brb3.3649 39169455 PMC11338839

[ref2] Baron Shahaf D. Weissman A. Priven L. Shahaf G. (2022). Identifying recall under sedation by a novel EEG based index of attention—a pilot study. Front. Med. 9:880384. doi: 10.3389/fmed.2022.880384, 35492350 PMC9047181

[ref3] Bart O. Liberman L. (2020). Sustained attention in exposure to tactile stimuli among children 4 to 10 years old with and without sensory modulation disorders. Am. J. Occup. Ther. 74, 7411505261p1–7411505261p1. doi: 10.5014/ajot.2020.74s1-po9802

[ref4] Bartur G. Joubran K. Peleg-Shani S. Vatine J. J. Shahaf G. (2020). A pilot study on the electrophysiological monitoring of patient’s engagement in post-stroke physical rehabilitation. Disabil. Rehabil. Assist. Technol. 15, 471–479. doi: 10.1080/17483107.2019.1680749 31684777

[ref5] Delorme A. Sejnowski T. Makeig S. (2007). Enhanced detection of artifacts in EEG data using higher-order statistics and independent component analysis. NeuroImage 34, 1443–1449. doi: 10.1016/j.neuroimage.2006.11.004, 17188898 PMC2895624

[ref6] den Hollander M. Goossens M. de Jong J. Ruijgrok J. Oosterhof J. Onghena P. . (2016). Expose or protect? A randomized controlled trial of exposure *in vivo* vs pain-contingent treatment as usual in patients with complex regional pain syndrome type 1. Pain 157, 2318–2329. doi: 10.1097/j.pain.0000000000000651 27429174

[ref7] den Hollander M. Smeets R. J. van Meulenbroek T. Laake-Geelen C. C. Baadjou V. A. Timmers I. (2022). Exposure *in vivo* as a treatment approach to target pain-related fear: theory and new insights from research and clinical practice. Phys. Ther. 102:pzab270. doi: 10.1093/ptj/pzab270 35084025

[ref8] Glombiewski J. A. Holzapfel S. Riecke J. Vlaeyen J. W. de Jong J. Lemmer G. . (2018). Exposure and CBT for chronic back pain: an RCT on differential efficacy and optimal length of treatment. J. Consult. Clin. Psychol. 86, 533–545. doi: 10.1037/ccp0000298, 29781651

[ref9] Goncharova I. I. McFarland D. J. Vaughan T. M. Wolpaw J. R. (2003). EMG contamination of EEG: spectral and topographical characteristics. Clin. Neurophysiol. 114, 1580–1159. doi: 10.1016/s1388-2457(03)00093-2 12948787

[ref10] Gvion A. Shahaf G. (2023). Real-time monitoring of barriers to patient engagement for improved rehabilitation: a protocol and representative case reports. Disabil. Rehabil. Assist. Technol. 18, 849–861. doi: 10.1080/17483107.2021.1929513, 34033726

[ref11] Gvion A. Stark R. Bartur G. Shahaf G. (2021). Behavioural and electrophysiological evaluation of the impact of different cue types upon individuals with acquired anomia. Aphasiology 35, 1519–1543. doi: 10.1080/02687038.2020.1822988

[ref12] Hedman-Lagerlöf M. Hedman-Lagerlöf E. Axelsson E. Ljótsson B. Engelbrektsson J. Hultkrantz S. . (2018). Internet-delivered exposure therapy for fibromyalgia: a randomized controlled trial. Clin. J. Pain 34, 532–542. doi: 10.1097/AJP.0000000000000566, 29077623

[ref13] Isserles M. Daskalakis Z. J. George M. S. Blumberger D. M. Sackeim H. A. Shahaf G. (2018). Simple electroencephalographic treatment-emergent marker can predict repetitive transcranial magnetic stimulation antidepressant response—a feasibility study. J. ECT 34, 274–282. doi: 10.1097/yct.0000000000000551, 30407932

[ref14] Karpin H. Misha T. Herling N. T. Bartur G. Shahaf G. (2022). Bedside patient engagement monitor for rehabilitation in disorders of consciousness–demonstrative case-reports. Disabil. Rehabil. Assist. Technol. 17, 539–548. doi: 10.1080/17483107.2020.1800112, 32730121

[ref15] Keren A. Fisher O. Hamde A. Tsafrir S. Ratzon N. Z. (2024). Reducing driving risk factors in adolescents with attention deficit hyperactivity disorder (ADHD): insights from EEG and eye-tracking analysis. Sensors 24:3319. doi: 10.3390/s24113319, 38894111 PMC11174634

[ref16] Kroll H. R. (2015). Exercise therapy for chronic pain. Phys. Med. Rehabil. Clin. 26, 263–281. doi: 10.1016/j.pmr.2014.12.007, 25952064

[ref17] Maffei A. Angrilli A. (2018). Spontaneous eye blink rate: an index of dopaminergic component of sustained attention and fatigue. Int. J. Psychophysiol. 123, 58–63. doi: 10.1016/j.ijpsycho.2017.11.009, 29133149

[ref18] Marris D. Theophanous K. Cabezon P. Dunlap Z. Donaldson M. (2021). The impact of combining pain education strategies with physical therapy interventions for patients with chronic pain: a systematic review and meta-analysis of randomized controlled trials. Physiother. Theory Pract. 37, 461–472. doi: 10.1080/09593985.2019.1633714, 31250682

[ref19] Orr S. P. Pitman R. K. Lasko N. B. Herz L. R. (1993). Psychophysiological assessment of posttraumatic stress disorder imagery in world war II and Korean combat veterans. J. Abnorm. Psychol. 102:152. doi: 10.1037//0021-843x.102.1.152, 8436691

[ref20] Palacios-García I. Silva J. Villena-González M. Campos-Arteaga G. Artigas-Vergara C. Luarte N. . (2021). Increase in beta power reflects attentional top-down modulation after psychosocial stress induction. Front. Hum. Neurosci. 15:630813. doi: 10.3389/fnhum.2021.630813, 33833671 PMC8021732

[ref21] Shahaf G. Kuperman P. Bloch Y. Yariv S. Granovsky Y. (2018a). Monitoring migraine cycle dynamics with an easy-to-use electrophysiological marker—a pilot study. Sensors 18:3918. doi: 10.3390/s18113918 30441751 PMC6263618

[ref22] Shahaf G. Nitzan U. Erez G. Mendelovic S. Bloch Y. (2018b). Monitoring attention in ADHD with an easy-to-use electrophysiological index. Front. Hum. Neurosci. 12:32. doi: 10.3389/fnhum.2018.00032, 29449806 PMC5799268

[ref23] Turk D. C. Wilson H. D. (2010). Fear of pain as a prognostic factor in chronic pain: conceptual models, assessment, and treatment implications. Curr. Pain Headache Rep. 14, 88–95. doi: 10.1007/s11916-010-0094-x, 20425197 PMC2872063

[ref24] Vasquez B. P. Lloyd-Kuzik A. Santiago A. T. Shahaf G. Lass J. W. (2023). Attentional engagement during mobile application skill learning among patients with memory impairment: a case series exploration. Am. J. Occup. Ther. 77:50064. doi: 10.5014/ajot.2023.050064, 36764006

[ref25] Woods M. P. Asmundson G. J. (2008). Evaluating the efficacy of graded in vivo exposure for the treatment of fear in patients with chronic back pain: a randomized controlled clinical trial. Pain 136, 271–280. doi: 10.1016/j.pain.2007.06.037 17716819

[ref26] Yogev-Seligmann G. Krasovsky T. Kafri M. (2022). Compensatory movement strategies differentially affect attention allocation and gait parameters in persons with Parkinson’s disease. Front. Hum. Neurosci. 16:943047. doi: 10.3389/fnhum.2022.943047, 36061510 PMC9433535

