# Supplementary figures and images for: Case Report: Introducing @tension—a system for improving adherence to exposure therapy in chronic pain patients
Source: Front Hum Neurosci. 2026 Feb 11;20:1708612. doi: 10.3389/fnhum.2026.1708612 (PMC12932578; doi:10.3389/fnhum.2026.1708612)

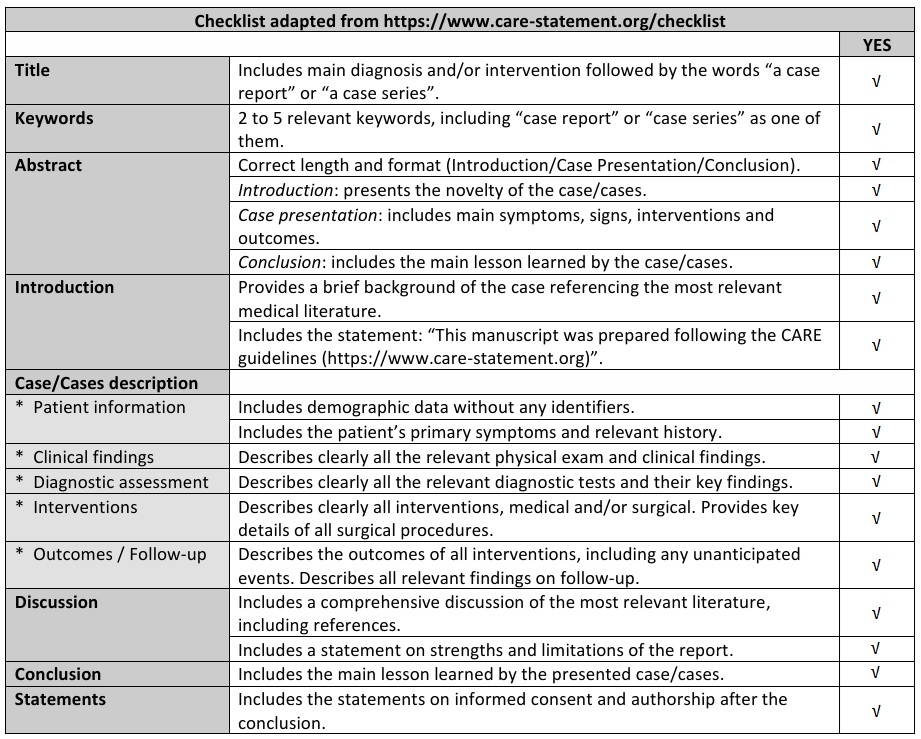

Supplement: Supplementary file 1 [file Image_1.JPEG]
